# SOD1-positive aggregate accumulation in the CNS predicts slower disease progression and increased longevity in a mutant SOD1 mouse model of ALS

**DOI:** 10.1038/s41598-019-43164-z

**Published:** 2019-04-30

**Authors:** Cindy Gill, James P. Phelan, Theo Hatzipetros, Joshua D. Kidd, Valerie R. Tassinari, Beth Levine, Monica Z. Wang, Andrew Moreno, Kenneth Thompson, Marcel Maier, Jan Grimm, Alan Gill, Fernando G. Vieira

**Affiliations:** 1 ALS Therapy Development Institute, Cambridge, Massachusetts, USA; 2 Neurimmune AG, Schlieren-Zurich, Switzerland

**Keywords:** Protein aggregation, Amyotrophic lateral sclerosis

## Abstract

Non-natively folded variants of superoxide dismutase 1 (SOD1) are thought to contribute to the pathogenesis of familial amyotrophic lateral sclerosis (ALS), however the relative toxicities of these variants are controversial. Here, we aimed to decipher the relationships between the different SOD1 variants (aggregated, soluble misfolded, soluble total) and the clinical presentation of ALS in the SOD1^G93A^ mouse. Using a multi-approach strategy, we found that the CNS regions least affected by disease had the most aggregated SOD1. We also found that the levels of aggregated SOD1 in the spinal cord were inversely correlated with the disease progression. Conversely, in the most affected regions, we observed that there was a high soluble misfolded/soluble total SOD1 ratio. Taken together, these findings suggest that soluble misfolded SOD1 may be the disease driver in ALS, whereas aggregated SOD1 may serve to sequester the toxic species acting in a neuroprotective fashion.

## Introduction

Amyotrophic lateral sclerosis (ALS) is a progressive neurodegenerative disease characterized by dysfunction and degeneration of lower motor neurons in the spinal cord and brain stem and upper motor neurons in the motor cortex^1,2^. When these neurons fail, the affected person experiences fasciculations, spasticity, weakness, muscle atrophy, and ultimately fatal paralysis^1^. ALS can be classified into two categories: familial ALS (FALS) and sporadic ALS (SALS) which account for approximately 10% and 90% of ALS cases, respectively^2^. SALS and FALS cases clinically present similarly, but FALS can be inherited^2^.

Multiple genes have been identified which, when mutated, can cause ALS in an autosomal dominant manner. Superoxide dismutase 1 (SOD1) mutations account for approximately 20% of FALS cases and 2% of all ALS cases^3^. SOD1 mutation association with FALS was first reported in 1993 when 11 different SOD1 missense mutations were identified in 13 different FALS families^4^. Since then, at least 140 mutations spanning all SOD1 protein domains have been associated with dominantly inherited FALS^5^.

SOD1 is an anti-oxidant enzyme that catalyzes the dismutation of superoxide radicals^6^. Both normal and mutated SOD1 exhibit physical characteristics that increase the likelihood of unfolding, misfolding, polymerization, aggregation, loss of function, and toxic gain of function^7–17^. Maturation of SOD1 to a functional enzyme is a complicated multistep process involving N-terminal acetylation, zinc ion insertion, chaperone-dependent copper ion insertion, formation of a disulfide bond between cysteines 57 and 146, and, finally, homodimerization. Failure at any of these steps can predispose SOD1 to misfold and/or polymerize. Additionally, SOD1 cysteine 111 (Cys111) is an exposed residue available for oxidative modification which promotes disulfide-bond-independent aggregation of SOD1^13^. Finally, human SOD1 exposes a tryptophan at residue 32 (Trp32) that provides an alternate site for SOD1 self-association leading to misfolding^9,11,14,18^.

Although understanding of the physicochemical properties of wild-type human SOD1 and its various mutations has continually improved since its identification as an ALS-causative risk factor, how SOD1 mutations actually cause motor neuron degeneration remains unclear. SOD1 loss of function may contribute to the neurodegeneration, but it is less likely to be the primary SOD1 FALS disease driver because some FALS SOD1 mutants seem to retain enzymatic activity and SOD1 null mice do not exhibit overt neurodegeneration^19–23^. It has been posited that misfolding and/or aggregation is the most likely source of SOD1 toxicity^5^. The large number and functional variety of SOD1 mutations leading to a common disease manifestation suggest that deviation from native wild-type SOD1 folding is a plausible candidate for mutation-based disease etiology^5^. In fact, multiple studies have revealed that many strains of mutant SOD1 and oxidized SOD1 can be toxic in cells and, more specifically, in neuronal lines^10–12,15,16,24–26^.

While there is general consensus that non-natively folded SOD1 is toxic, the roles of SOD1 aggregation in ALS disease pathogenesis remain controversial. SOD1-positive inclusion bodies and aggregates have been observed consistently in post-mortem CNS of patients with SOD1 FALS^27–29^. Further, SOD1-positive inclusion bodies and misfolded SOD1 have been detected in some cases of SALS^27,30–33^. In addition, SOD1-positive aggregates are often observed in cell-based and murine models of SOD1-mediated cell death and ALS^34–39^. Some have suggested that spatially organized aggregation of misfolded proteins is a cellular stress defense strategy^40^. In one recent paper, genetic disruption of lysosomal targeting to autophagic substrates increased SOD1 aggregates robustly in SOD1^G93A^ mice, but only moderately affected the phenotype^41^. However, while most of these studies show a correlation between SOD1 aggregation and neurodegeneration, few have generated any data to elucidate whether the SOD1 aggregation process is a disease driver, a protective compensatory mechanism, or a combination of the two scenarios.

Many of the questions that arise about the roles of misfolded and aggregated SOD1 in ALS are mirrored in other neurodegenerative diseases. Correlations between protein aggregation in the central nervous system and many neurodegenerative diseases including Alzheimer’s, Parkinson’s, and Huntington’s diseases have been recognized^41,42^. However, the complexities of the diseases and the dynamic nature of protein aggregation have made it very difficult to establish whether aggregation is pathogenic, protective, or both, depending on the type and context^41,42^.

Here, we report on a series of experiments exploring SOD1 protein aggregation using B6-SJL-Tg(SOD1*G93A)1Gur/J mice (SOD1^G93A^ mice). The studies take advantage of recently described human monoclonal antibodies (α-miSOD1 and NI204.P) developed by Neurimmune with very high affinities for misfolded human SOD1, but low affinities for properly folded mature human SOD1^33^. The current studies were designed to shed light on the relationships between large SOD1 positive aggregates (aggSOD1), soluble misfolded SOD1 (s-misSOD1), total soluble human SOD1 (s-humSOD1), mouse clinical symptom presentation, and individual mouse clinical outcomes and longevity.

## Results

### Spinal Cord Immunohistochemistry

**Figure 1:**
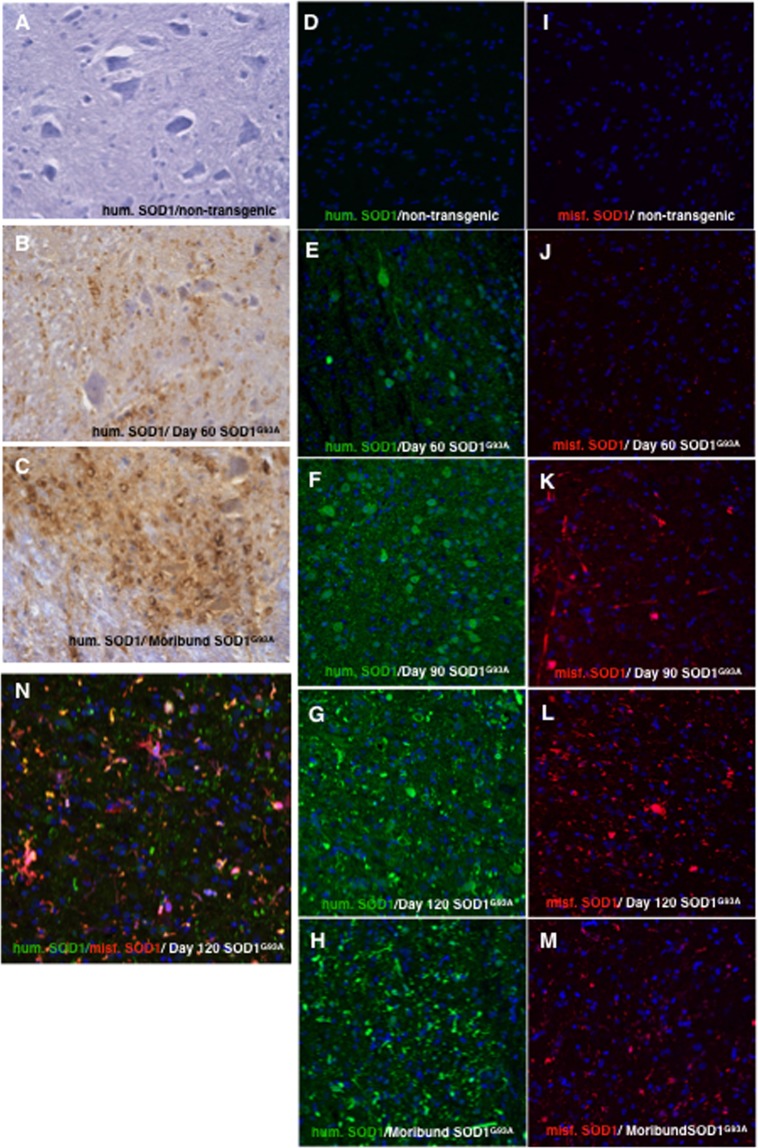
Immunohistochemistry and immunofluorescence of human SOD1 in lumbar spinal cord ventral horn of non-transgenic mice and in mice expressing mutant human SOD1. (**A**–**C**) Immunostaining of spinal cord using a polyclonal antibody reagent raised against human SOD1 (ab52950) with secondary antibody conjugated to DAB peroxidase: (**A**) Spinal cord from nontransgenic mouse with negligible background, (**B**) presymptomatic SOD1^G93A^ spinal cord, (**C**) moribund SOD1^G93A^ spinal cord. (**D**–**H**) Immunofluorescence staining of spinal cord using anti-human SOD1 (ab52950) polyclonal antibody (green = human SOD1, blue = DAPI): (**D**) non-transgenic mouse with negligible background, (**E**) presymptomatic SOD1^G93A^ spinal cord, (**F**) early symptomatic SOD1^G93A^ spinal cord, (**G**) late symptomatic SOD1^G93A^ spinal cord, (**H**) moribund SOD1^G93A^ spinal cord. (**I**–**M**) Immunofluorescence staining of spinal cord using a human antibody (NI204.P) with high specificity for misfolded human SOD1 (red = misfolded human SOD1, blue = DAPI): (**I**) non-transgenic mouse with negligible background, (**J**) presymptomatic SOD1^G93A^ spinal cord, (**K**) early symptomatic SOD1^G93A^ spinal cord, (**L**) late symptomatic SOD1^G93A^ spinal cord, (**M**) moribund SOD1^G93A^ spinal cord. (**N**) Double–labeled immunofluorescence of both total human SOD1 (green) and misfolded human SOD1 (red) of late symptomatic SOD1^G93A^ mouse spinal cord.

Immunohistochemical (IHC) and immunofluorescence (IF) measurement of SOD1 in the lumbar spinal cord of SOD1^G93A^ mice was performed in 3 to 4 male mice at each time point (panel) represented in Fig. 1; each mouse is represented individually in Supplemental Fig. 1 through 6. Lumbar spinal cord was the focus of these studies because this is the region of the central nervous system with which clinical symptoms of SOD1^G93A^ mice are most closely associated^23^.

IHC staining of transgenically-expressed human SOD1 in ventral horn regions of lumbar spinal cord from pre-symptomatic and end-stage SOD1^G93A^ mice revealed widespread expression of the human SOD1 transgene that increased with age (Fig. 1B,C) and was absent in non-transgenic littermates (Fig. 1A). The findings replicated observations by Jaarsma, *et al*. in 2000, confirming the appearance of human SOD1-positive vacuoles in spinal ventral grey columns and in the ventrolateral funiculi^43^. In our studies, these SOD1-positive vacuoles were larger in moribund mice (Fig. 1C) than in presymptomatic mice (Fig. 1B). Jaarsma, *et al*. colocalized these SOD1 positive vacuolar structures with cytochrome C and, in conjunction with electron microscopy, concluded that these structures were primarily swollen mitochondria in neuronal dendrites and axons^43^.

IF studies using the same rabbit polyclonal primary antibody (ab52950) as the one used in the IHC studies similarly revealed age-correlated increases in detectable human SOD1 in lumbar spinal cord ventral horns from SOD1^G93A^ mice, which were absent in non-transgenic littermates (Fig. 1D–H, Suppl. Figs 1–5). The vacuolar features were less easily observed when using IF (Fig. 1E–H) techniques than when using IHC (1B,C).

The aforementioned IHC and IF approaches for detection of total human SOD1 were unable to reveal how much of the detectable human SOD1 was either misfolded or aggregated. For this reason, sections from the same mice were probed for misfolded human SOD1 using a specific anti-misfolded human SOD1 antibody called NI204.P, shared with us by Neurimmune^33^. A markedly different staining pattern was observed than that revealed by IHC/IF detecting total human SOD1 (Fig. 1I–M). Unlike total SOD1 (Fig. 1B–G), misfolded SOD1 appeared more enriched in motor neurons of SOD1^G93A^ mice (Fig. 1J–N, Suppl. Figs 1–5). Like total SOD1, misfolded SOD1 detection increased from presymptomatic P60 to overtly symptomatic P120 (Fig. 1I–M). As expected, no human SOD1 (Fig. 1D) or misfolded human SOD1 (Fig. 1I) was detected in non-transgenic littermates of the SOD1^G93A^ mice in these studies (Suppl. Fig. 1). Total human SOD1 staining revealed higher frequencies of larger intensely-stained puncta at P120 and when the mice were moribund (Fig. 1G,H). Small foci of misfolded SOD1 were detected in the spinal cord of presymptomatic P60 SOD1^G93A^ mice (Fig. 1J). Spinal cord ventral horns from P90 early symptomatic SOD1^G93A^ mice revealed misfolded SOD1 located in motor neurons and in axons (Suppl. Fig. 3). In later symptomatic P120 SOD1^G93A^ mice, misfolded SOD1 staining revealed more frequent bright puncta (Fig. 1L, Suppl. Fig. 4), similar to those observed when the same mice were stained for total SOD1 (Fig. 1G). Spinal cord sections from P120 SOD1^G93A^ mice stained for both total and misfolded SOD1 revealed consistent, but incomplete, colocalization of total human SOD1 puncta and misfolded SOD1 puncta (Fig. 1N).

**Figure 2:**
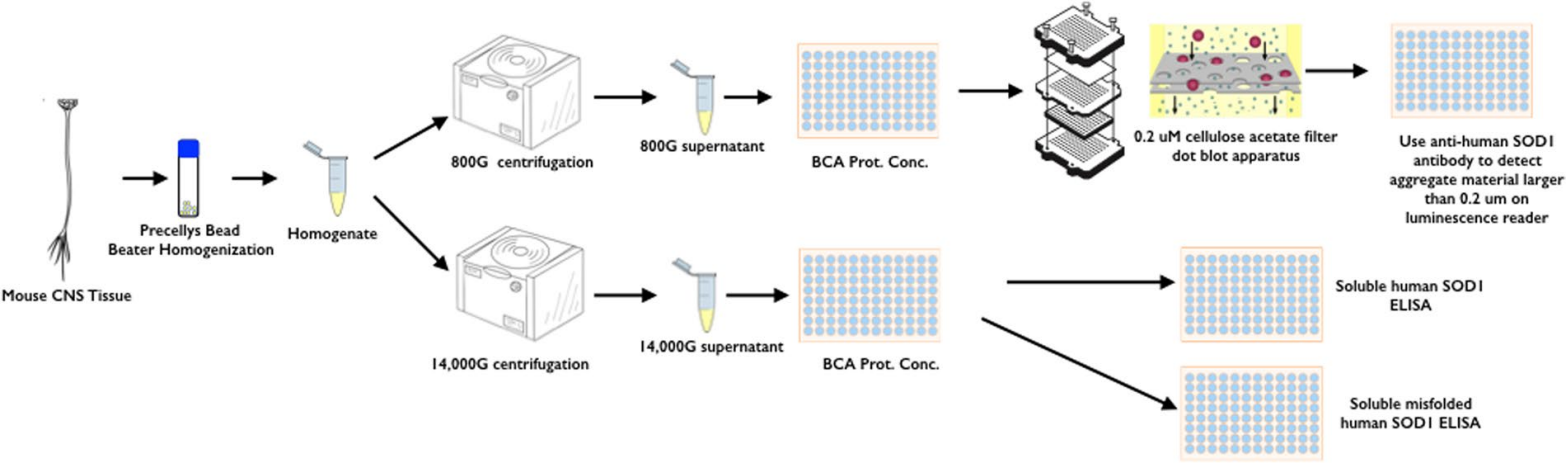
Central nervous system tissue sample workflow for quantitation of total soluble human SOD1, soluble misfolded human SOD1, and SOD1 positive aggregates larger than 0.2 microns.

To neutralize the effect of non-specific staining, immunohistochemistry and immunofluorescence protocols were benchmarked against negative control staining using normal mouse serum in place of primary antibody (Suppl. Fig. 6). While very informative, IHC/IF methods ultimately have limitations. In particular, it is impossible to discern whether the SOD1 or misfolded SOD1 detected have formed into aggregated inclusions. Similarly, with no standard curves against which to compare signals, it is impossible to quantitate the total SOD1, misfolded SOD1, and aggregated SOD1 (either natively folded or misfolded). To shed light on the relative contributions of these SOD1 variants to pathology and disease in the SOD1^G93A^ mice, protocols were developed for detection and quantitation of total soluble human SOD1 (s-humSOD1), misfolded soluble SOD1 (s-misSOD1), and aggregated SOD1 (aggSOD1) (Fig. 2). Here, we concentrated first on spinal cords from SOD1^G93A^ mice that were 30, 60, 90, 120, or 130 days old.

### Total Soluble Human SOD1

**Figure 3:**
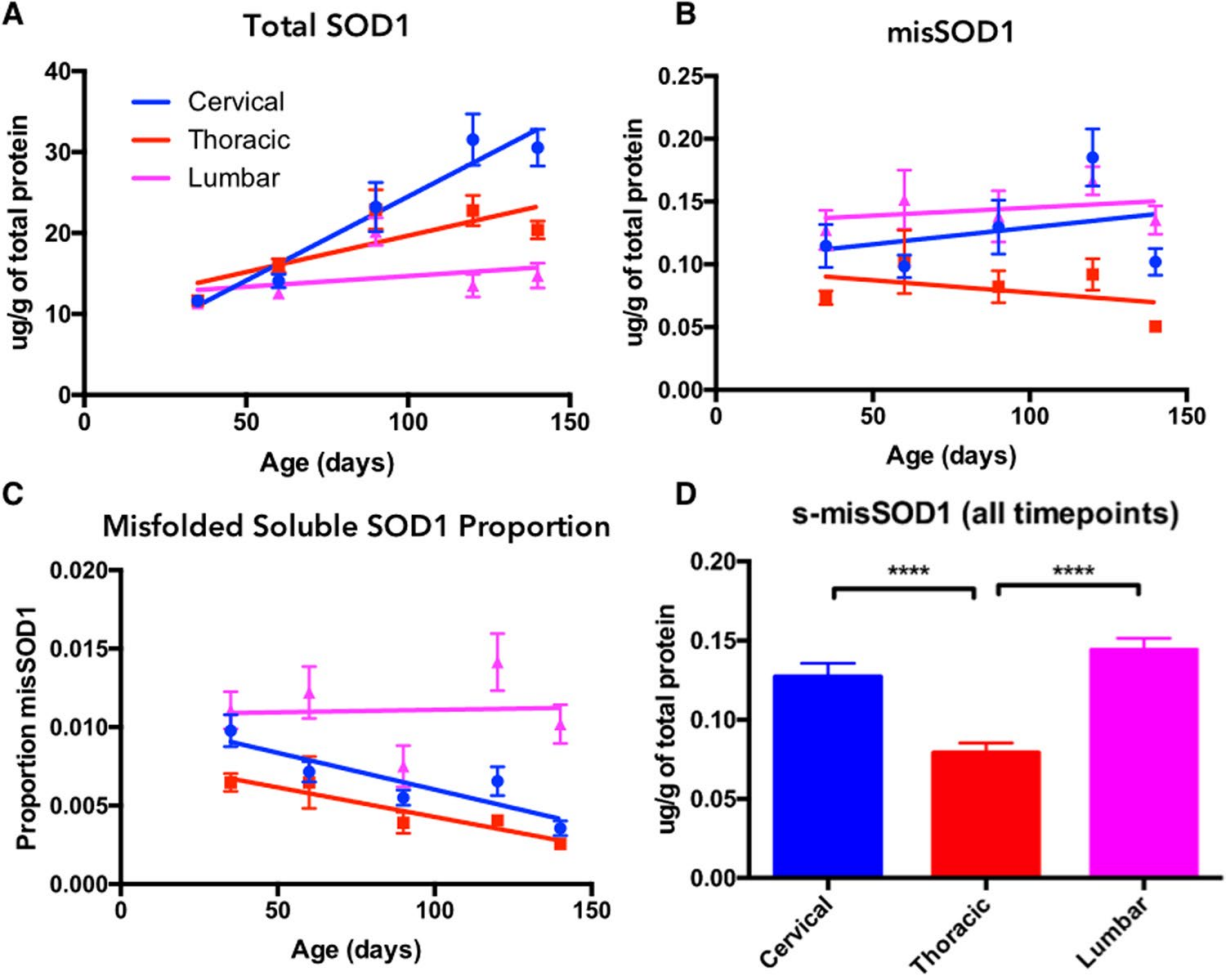
(**A**) Total soluble human SOD1 in three regions of SOD1^G93A^ mouse spinal cord over time. (**B**) Soluble misfolded human SOD1 in three regions of SOD1^G93A^ mouse spinal cord over time. (**C**) The proportion of total soluble human SOD1 that was measured as misfolded in three regions of SOD1^G93A^ spinal cord over time. (**D**) Soluble misfolded SOD1 in each region of spinal cord at all time points.

Spinal cord samples were divided into cervical, thoracic, and lumbar regions. Using an ELISA, s-humSOD1 was quantitated in detergent-free supernatants of tissue homogenates that were spun at 14,000 × *g*. S-humSOD1 increased significantly in cervical (R^2^ = 0.47, p < 0.0001) and thoracic (R^2^ = 0.2566, p < 0.0001) spinal cord of SOD1^G93A^ mice, but not in lumbar spinal cord (Fig. 3A, Table 1) (P35 n = 12, P60 n = 11, P90 n = 12, P120 n = 14, Moribund n = 14).

**Table 1:** Correlation statistics for soluble misfolded SOD1, soluble total SOD1, and misfolded SOD1 proportion vs. mouse age in cervical, thoracic, and lumbar spinal cord.

		Spinal Cord Soluble SOD1 Change over Time				
Spinal Cord Region	Soluble Total SOD1		Soluble Misfolded SOD1		Misfolded SOD1 Proportion	
	R^2^	P value	R^2^	P value	R^2^	P value
Cervical	0.4787	<0.0001	0.0229	0.2327	0.3012	<0.0001
Thoracic	0.2566	<0.0001	0.0247	0.2191	0.2250	<0.0001
Lumbar	0.0358	0.1374	0.0071	0.5096	0.0005	0.8652

### Soluble Misfolded Human SOD1

The same lysates assayed for s-humSOD1 were also assayed in an ELISA using a primary antibody selective for misSOD1 (courtesy of Neurimmune)^33^. Unlike s-humSOD1, s-misSOD1 did not significantly increase with age in any of the spinal cord regions (Fig. 3B). Thoracic spinal cord harbored significantly less than both lumbar and cervical spinal cord (Fig. 3D).

An assessment of the proportion of s-humSOD1 that was misfolded revealed a significant decrease over time in both cervical (R^2^ = 0.3012, p < 0.0001) and thoracic (R^2^ = 0.2250, p < 0.0001) spinal cord, which was not evident in lumbar spinal cord (Fig. 3C). In addition, the proportion of s-misSOD1 in lumbar spinal cord across all time points was significantly elevated compared to either cervical or thoracic spinal cord from the same mice (Suppl. Fig. 7).

### SOD1 mRNA Expression in Spinal Cord

**Figure 4:**
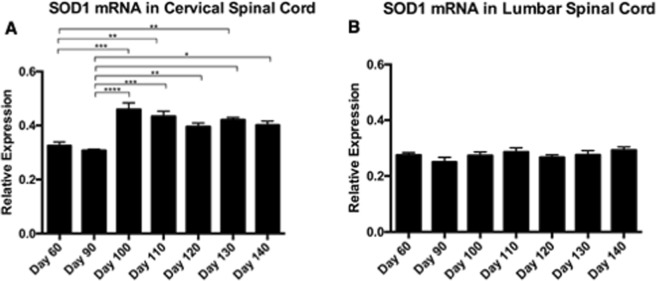
Relative SOD1 mRNA expression in spinal cord of SOD1^G93A^ mice: (**A**) Relative SOD1 mRNA expression in cervical spinal cord at P60, 90, 100, 110, 120, 130, and 140 days. (**B**) Relative SOD1 mRNA expression in lumbar spinal cord at P60, 90, 100, 110, 120, 130, and 140 days.

To determine whether age-dependent changes in total soluble SOD1 protein levels in cervical spinal cord were at least partially explainable by increased transgene mRNA expression, cervical and lumbar spinal cord samples were assayed by qPCR and compared (P60 n = 4, P90 n = 4, P100 n = 4, P110 n = 8, P120 n = 11, P130 n = 7, P140 n = 6). SOD1 mRNA expression in cervical spinal cord increased significantly at P100 (Fig. 4A). No comparable change was observed in lumbar spinal cord (Fig. 4B). This suggested that increased total soluble SOD1 in cervical spinal cord may partly be explained by increased mRNA expression.

### SOD1 Aggregate

**Figure 5:**
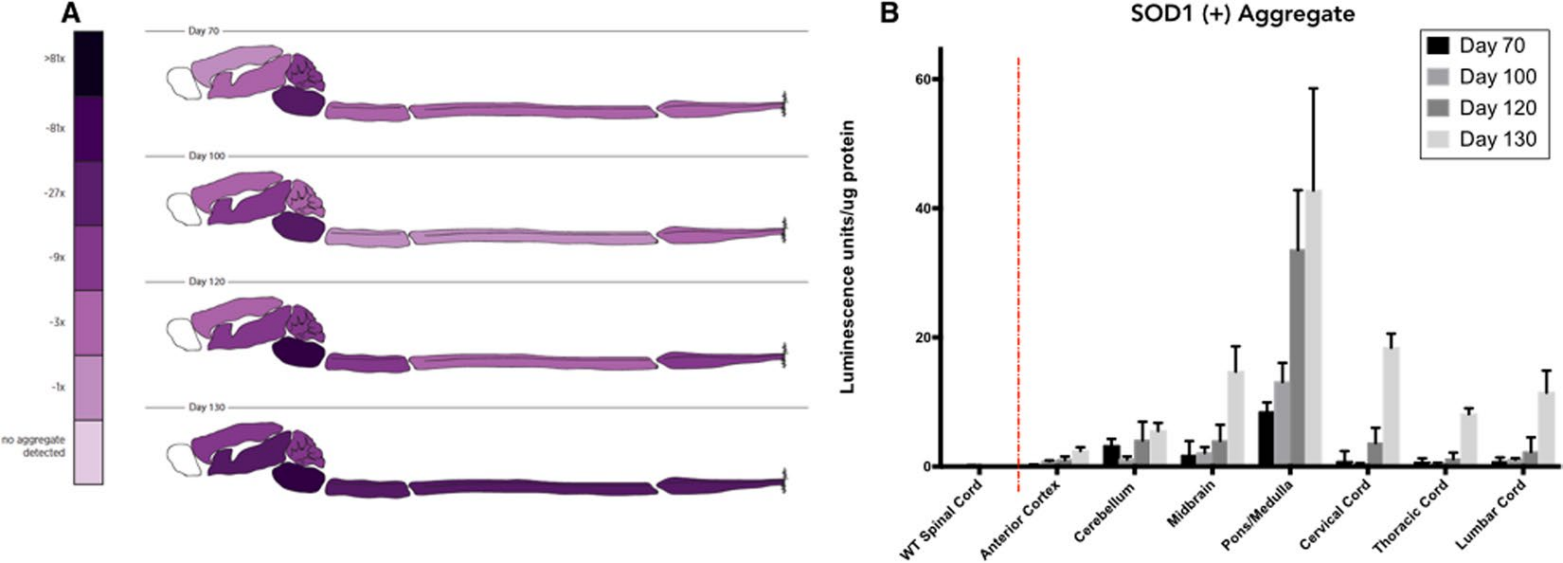
Human SOD1 protein aggregate in central nervous system of SOD1^G93A^ mice: (**A**) Pictographic representation of relative SOD1 positive aggregate load in the central nervous system of SOD1^G93A^ mice at age 70, 100, 120, and 130 days. (**B**) Bar graph of relative SOD1 positive aggregate load in seven anatomical regions of SOD1^G93A^ mouse central nervous system.

The lack of an appreciable increase in s-humSOD1 and s-misSOD1 in the lumbar spinal cord was inconsistent with the IHC and IF staining (Fig. 1B–K). This suggested the possibility that the visible increases in SOD1 might be sequestered in larger, insoluble aggregates. To study relative aggSOD1 load, a filter retardation assay was developed (Fig. 2). Generally, the assay system applied supernatants that were collected from homogenates after an 800 × *g* centrifugation step that separated macroscopic fibrous debris while permitting higher molecular weight protein aggregates to remain suspended in the supernatant. The samples were normalized for total protein concentration, then loaded onto a 0.2 μm pore cellulose acetate membrane within a 96-well vacuum filtration apparatus. After vacuum filtration, only material with diameters greater than 0.2 μm were trapped. The cellulose acetate membrane was then probed with an anti-human SOD1 antibody. SOD1-positive aggregates were detected using a chemiluminescent substrate in a 96-well luminescence plate reader. All relative aggSOD1 data presented in Fig. 5 came from a single assay run to ensure reliable interpretation of relative aggregate load.

In this study, in addition to cervical, thoracic, and lumbar spinal cord, subdivisions of brain including anterior cerebral cortex, midbrain, pons/medulla, and cerebellum were also assayed. The time points included were age days 70, 100, 120, and 130. The pons/medulla yielded the most intense aggregate signal at all time points (Fig. 5A,B). This was consistent with reports that the most prominent SOD1 staining in late stage SOD1^G93A^ mice is observed in the pons and medulla^44^. Without exception, all tissue types assayed demonstrated highest aggregate load at P130, significantly so in anterior cortex, midbrain, cervical spinal cord, thoracic spinal cord, and lumbar spinal cord (Fig. 5A,B, Suppl. Tables 1–7). Overall, pons/medulla harbored the most aggSOD1 (Fig. 5A,B, Suppl. Table 8).

In the lumbar spinal cord region, there was no significant increase in either s-humSOD1 (Fig. 3A) or in s-misSOD1 (Fig. 3B), and so the s-misSOD1/s-humSOD1 ratio remained stable (Fig. 3C, Table 1). Yet there was an increase in aggSOD1 (Fig. 5A,B, Suppl. Table 7). This contrasts with cervical and thoracic spinal cord regions, where despite marked increases in aggSOD1 with age, the s-misSOD1/s-humSOD1 ratio decreased (Fig. 3C, Table 1).

### SOD1 Aggregate vs. Disease Symptom Progression

Unlike s-humSOD1 and s-misSOD1, aggSOD1 increased in all three spinal cord tissue regions with age. An experiment was designed to test the hypothesis that early increases in aggSOD1 will predict more aggressive disease progression. To do this, mice (n = 29, Table 2) were assigned to cohorts where termination and spinal cord sample collection were dictated by the first instance of three discrete symptomatic presentations defined by a previously described neurological scoring system, NeuroScore (NS)^45^. Briefly, NeuroScore is an ordinal scale ranging from 0 to 4 where higher scores represent greater degrees of motor impairment in SOD1^G93A^ mice. For this experiment, we concentrated on NS 2, 3, and 4, representing onset of hindlimb paresis, complete hindlimb paralysis, and moribundity, respectively. Each mouse had a predefined termination point at a score of 2, 3, or 4.

**Table 2:** Correlation statistics for SOD1 positive aggregate load in whole spinal cord vs. overall age and at specific symptomatic stages in SOD1^G93A^ mice.

	SOD1 Positive Aggregate Load vs Neurological Stage at Termination			
Neurological Stage	n	Slope	R^2^	P value
Paresis Onset	10	21.5 +/− 17.1	0.1647	0.2450
Hind-Limb Paralysis	8	107.5 +/− 7.9	0.9689	<0.0001
Moribund	11	112.1 +/− 36.8	0.5084	0.0138
Overall	29	39.3 +/− 13.8	0.2242	0.0080

**Figure 6:**
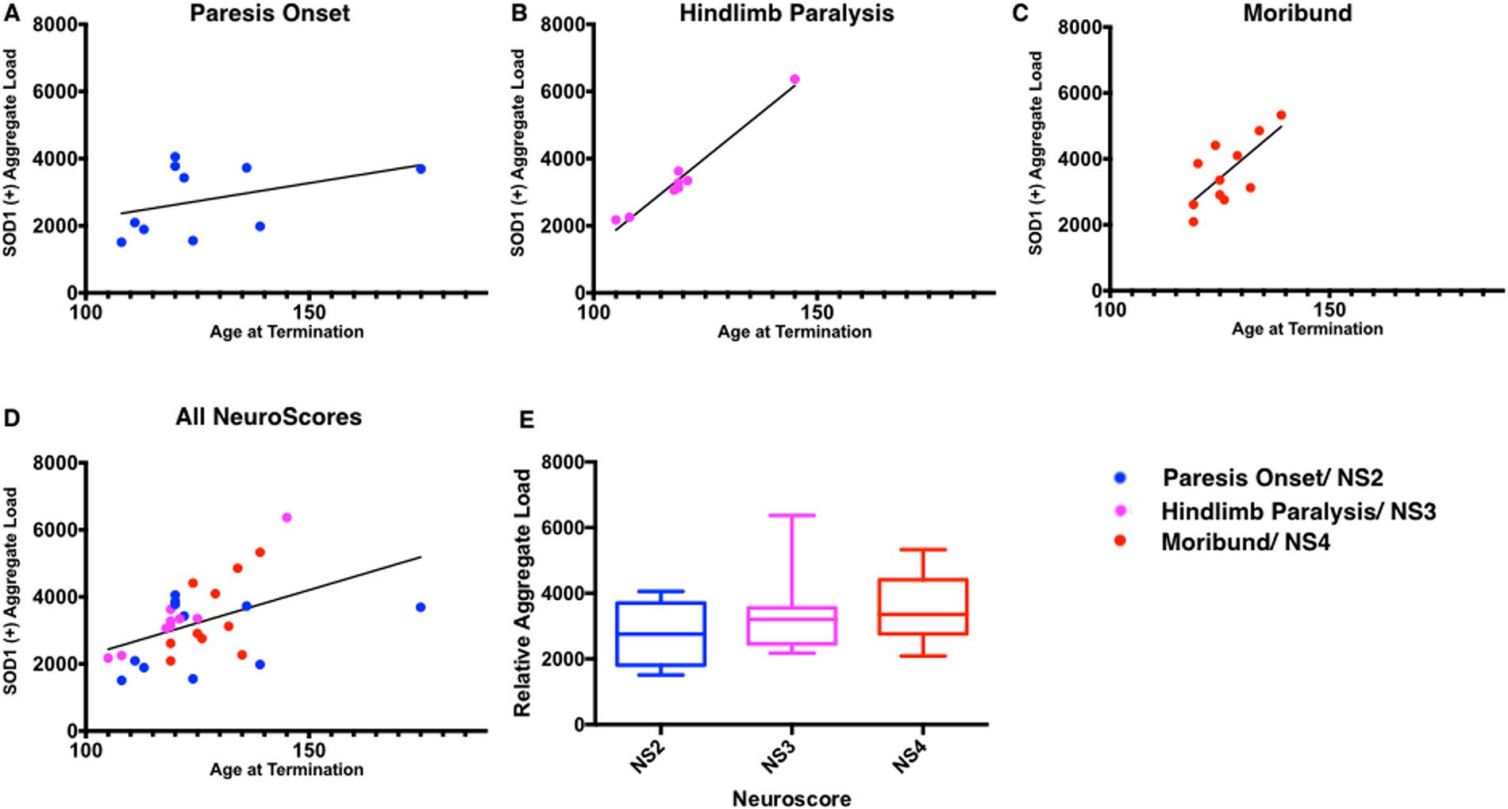
Relative SOD1 positive aggregate in whole spinal cord of SOD1^G93A^ mice vs. age at termination. (**A**) Relative aggSOD1 vs. age at termination (onset NS2 only). (**B**) Relative aggSOD1 vs. age at termination (onset NS3 only). (**C**) Relative aggSOD1 vs. age at termination (NS4/moribund only). (**D**) Relative aggSOD1 in whole spinal cord of SOD1^G93A^ mice vs. age at termination (all mice). (**E**) aggSOD1 load at three discrete symptomatic clinical presentations.

In SOD1^G93A^ mice, ages at specific NS can vary. This variation, while problematic in the context of drug testing, is an advantage when trying to determine which features of their motor neuron disease associate more closely with disease progression and which associate more closely with age. SOD1^G93A^ mice showed a definite correlation between their age and aggSOD1 loads in spinal cord (Figs 5A,B, 6D). However, there were no significant differences in aggregate loads present in spinal cords of mice at each of the three NeuroScores evaluated (Fig. 6E). There were very strong relationships between age and SOD1-positive aggregate loads within mice presenting with equivalent degrees of impairment (Fig. 6B,C, Table 2). Simply stated, mice with slower disease progression accumulated greater amounts of aggSOD1.

## Discussion

Protein aggregates are a feature common to many neurodegenerative diseases^46^. Strategies for inhibition of protein aggregate formation^46,47^ and disruption of existing protein aggregates^48^ are currently in development for multiple neurodegenerative diseases. However, there is growing recognition that all aggregates are not created equal. It is becoming increasingly evident that the heterogeneity of aggregates ranges from relatively small and unstructured oligomers to highly ordered and larger cross-beta-sheet amyloid fibrils^48,49^. Additional consideration has been directed toward understanding the various roles of aggregates and inclusions associated with intracellular organelles or functional proteins^50,51^.

In the majority of ALS cases, ubiquitinated protein aggregates are found in central nervous system tissues^51^. Interestingly, in ALS, protein inclusions occur not only in motor cortex and spinal cord, but also in tissues where neurodegeneration is less obvious in ALS. These include temporal cortex, hippocampus, and cerebellum^44^. Furthermore, in genetically associated ALS cases involving C9ORF72 dipeptide repeat protein (DRP), inclusions are extremely rare in spinal cord overall and almost absent from motor neurons specifically^52^. The dearth of DRP inclusions in the vulnerable cell population can be interpreted to suggest that the dipeptide repeat proteins do not cause degeneration^52^. An alternate interpretation is that the inability of those cells to sequester DRPs into detectable inclusions results in increased vulnerability. In SOD1^G93A^ mice we observe significantly more SOD1-positive aggregates in less impaired brain regions than in the more impaired regions of spinal cord, suggesting a lesser role for protein aggregates in motor neuron death (Fig. 5A,B).

We extend these observations to show that higher levels of aggregated SOD1 were found to be associated with slower disease progression. For example, spinal cord lysate from a mouse that became completely paralyzed at P145 had a three-fold greater aggregate load than a mouse that was equivalently impaired at P105 (Fig. 4B). At a minimum, these results suggest that SOD1^G93A^ mice can suffer terminal impairment while harboring a spinal cord aggregate load well below a maximum capacity. Going one step further, these data might suggest that the detected aggregates are neuroprotective.

Analyses of the soluble SOD1 fraction of lysates prepared from SOD1^G93A^ mouse spinal cord regions may further inform interpretation of the surprising SOD1 aggregate results. Motor neuron death is most prominent in the lumbar spinal cord of SOD1^G93A^ mice with the most severe symptoms associated with lumbar myotomes^23^. Surprisingly, levels of s-humSOD1 in lumbar spinal cord were lower and remained constant compared to cervical and thoracic regions where levels increased significantly with age (Fig. 3A). Conversely, we detected a greater proportion of s-misSOD1 per unit of s-humSOD1 in lumbar spinal cord (Fig. 3B,C, Suppl. Fig. 7). These findings may implicate s-misSOD1 as a disease driver in the more impaired lumbar spinal cord, with SOD1 aggregates serving to sequester the toxic species. This hypothesis is supported by recent observations that smaller non-native SOD1 oligomers are toxic to cells^15,16^.

It is also possible that the monotonic increases in s-humSOD1 levels in cervical and thoracic spinal cord that are missing in lumbar spinal cord are protective, given the delayed and less severe disease symptoms associated with cervical and thoracic myotomes^23^. Assessment of relative SOD1 transgene mRNA expression points to increasing expression of SOD1 in cervical spinal cord with age, a finding which is not evident in lumbar spinal cord in SOD1^G93A^ mice. The possibility that increased levels of properly folded, functional human SOD1 may be protective in transgenic mouse models of ALS is consistent with recent findings showing that SOD1 levels increase in a mouse model of SOD1 ALS following treatment with the efficacious agent, CuATSM^53^. In summary, our findings are consistent with a model where increased soluble human SOD1 is protective, soluble misfolded SOD1 is deleterious, and large SOD1 aggregates are either benign or protective.

While these findings shed light on possible behaviors of protein aggregates in the general context of neurodegeneration and of misfolded SOD1 in the specific context of ALS, SOD1^G93A^ mice, like all research models, have inherent limitations. Perhaps most notably, for the purposes of the current studies, is the fact that SOD1^G93A^ mice harbor more than 20 copies of the mutant human SOD1 transgene and are driven to over-express the protein. It may be that the SOD1 over-expression accelerates the observations described in this report, however it is not likely that over-expression is the main driver. After all, misfolded SOD1 has been widely observed in human patients carrying a single copy of mutant SOD1. SOD1 inclusions have been widely reported in SOD1 FALS and, though the point is controversial, misfolded SOD1 has been observed in SALS spinal cord and brain. Our observations will likely inform the interpretation of results from ongoing drug development programs aiming to suppress SOD1 expression or targeting misfolded SOD1 using antibodies.

Cellular mechanisms underlying our observations remain largely unclear. Generally, cellular proteostasis is maintained by the complex interplay of multiple intracellular pathways that affect the conformation, quantity, subcellular localization, function, and toxicity of various aberrantly modified SOD1 species. These pathways include chaperone-mediated protein refolding, protein degradation through either the ubiquitin-proteasome or autophagy pathways, and protein aggregation mediated through various distinct processes with equally distinct cellular outcomes^39,41,50^. Of the cellular processes engaged to deal with mutant human SOD1 in SOD1^G93A^ mice, we propose that some form of SOD1 aggregation is associated with the best clinical outcomes. Our findings support the hypothesis that a higher capacity to draw s-misSOD1 into aggregates translates into more resiliency against its toxicity. These results in a mammalian whole animal model of ALS are consistent with recent neuroblastoma cell results indicating that lower molecular weight non-native SOD1 trimers were cytotoxic, whereas larger SOD1 aggregates did not reduce cell viability^16^. Together, these results suggest that a characteristic of less resilient cells, tissues, and mice is to permit s-misSOD1, perhaps in the form of non-native SOD1 trimers, to directly injure motor neurons. Injury might occur through a poorly understood toxic gain-of-function or by engaging alternative stress responses that ultimately result in motor neuron dysfunction and death.

## Methods

### Animals

All the mice used in these experiments came from ALS TDI’s SOD1^G93A^ mouse colony. This colony was derived from the high copy B6SJLTgN(SOD1G93A)1Gur strain originally produced by Gurney, *et al*. (1994). The colony was maintained at Biomedical Research Models (Worcester, MA) by crossing transgenic C57BL/6-SJL males with wild-type C57BL/6-SJL F1 females. The F1 animals were generated by crossing SJL males with B6 females. Mice were shipped to ALS TDI at 35–45 days of age and were allowed at least one week to acclimate to the animal facility (12-hr light/dark cycle at a temperature of 18–23 °C and 40–60% humidity) before they were assigned to a study. Male SOD1^G93A^ mice were housed one mouse per cage, since group housed males tend to fight, while female SOD1^G93A^ mice were housed up to two per cage. Environmental enrichment was provided in the form of plastic huts. Food and water were provided *ad libitum*. All experiments were conducted in accordance with the protocols described by the National Institutes of Health Guide for the Care and Use of Animals and were approved by ALS TDI’s institutional animal care and use committee (IACUC).

Mice were housed in static isolator cages. Cages were changed weekly. Food and bedding was purchased from Envigo, formerly Harlan-Teklad. The bedding, Sani-chips, was a hardwood based product. The food, Teklad global 2918, was an irradiated 18% protein rodent diet. Water was filtered through a Millipore reverse osmosis system.

All animal holding rooms in our facility were spot checked at the beginning and end of each weekday and once on weekends. During each spot check, the animal was observed in the cage. Temperature and humidity were recorded on room door sheets located at the entrance of every holding room. Any animal health concerns, beyond normal phenotype expression, were also recorded on the door sheets and brought to the attention of the principle investigator and veterinary staff.

### Neurological Scoring (NeuroScore)

- NeuroScore is a phenotypic screening protocol focused on hindlimb function and was designed to provide an unbiased assessment of disease onset, progression and severity of paralysis (Hatzipetros *et al*., 2015). The NeuroScore protocol was observed daily by a trained experimenter who assigned NeuroScores (NS) for each hindlimb (left or right) independently based on the following set of observations on a 0–4 scale:
- NS 0 (Presymptomatic): When the mouse was suspended by the tail, the hindlimb presented a normal splay, *i.e*., it was fully extended away from the lateral midline and it stayed in this position for 2 sec or longer.
- NS 1 (First symptoms): When the mouse was suspended by the tail, the hindlimb presented an abnormal splay, *e.g*., it was collapsed, partially collapsed towards lateral midline, trembled, retracted/clasped.
- NS 2 (Onset of paresis): When the mouse was suspended by the tail, the hindlimb was partially or completely collapsed. When the mouse was allowed to walk, the hindlimb was used for forward motion however the toes curled downwards at least twice during a 90 cm walk, or any part of the foot dragged along the cage bottom/table. When the mouse was placed on its left and right side, it was able to right itself within 10 sec from both sides.
- NS 3 (Paralysis): When the mouse was suspended by the tail, there was rigid paralysis in the hindlimb or minimal joint movement. When the mouse was allowed to walk, there was forward motion, however the hindlimb was not being used for forward motion. When the mouse was placed on its left and right side, it was able to right itself within 10 sec from both sides.
- NS 4 (Humane end-point): When the mouse was suspended by the tail, there was rigid paralysis in the hindlimbs. When the mouse was allowed to walk, there was no forward motion. When the mouse was placed on its left AND right side, it was not able to right itself within 10 sec from either side.

### Tissue Extraction and Dissection

#### Spinal Cord Dissection

Mice were killed at different time-points, based on the experimental design, by carbon dioxide asphyxiation. Spinal cords and brains were rapidly excised, dissected, weighed, quick-frozen with liquid nitrogen, and stored at −80 °C. Spinal cord dissection was performed using one of two methods. The first method was performed in order to keep the entire length of the spinal cord intact. The head was removed with a single cut directly through the cervical vertebrae. A lumbar vertebra was cut directly above the iliac crests to expose the spinal cord rostrally and caudally. An 18-gauge needle was inserted into the caudal opening until snuggly fit. Then sterile saline was forced into the vertebral column in order to extrude the spinal cord into a weighing dish. The spinal cord was then separated longitudinally into the right and left sides using forceps and then quickly frozen on dry ice in a collection vial (Corning 430488). The second method was employed to orient the spinal cord into left and right halves and also to dissect the spinal cord into three distinct anatomical regions (lumbar, thoracic, cervical) for study. The skull was separated from the spinal cord by manually locating where the spinal cord connects at the base of the skull and making one cut straight through the spinal cord. The lumbar cord was cut directly above the iliac crests. The same needle procedure was performed to extrude the spinal cord. Orientation of anterior and posterior was determined by finding a dark pair of lines located only on the dorsal side of the spinal cord indicating the posterolateral sulci. Once oriented to have the dorsal side up, the change in width of the spinal cord was used as a reference to further dissect it into cervical, thoracic, and lumbar regions. Finally, each section was separated by forceps into left and right halves and quickly frozen in a collection vial on liquid nitrogen.

#### Brain Dissection

The base of the skull was located and one single cut was used to separate it from the body. Scissors were used to carefully remove a region of the skull by cutting in each direction from the exposed posterior-most point of the occipital bone to the lateral-most point of the occipital bone, then along to the posterior-most points of orbit on the dorsal view along both sides of the skull. Cuts were made forward to the sutures between the maxillary and frontal bones in the lachrymal region of the skull. The top of the skull was folded toward the anterior to expose the brain. Cranial nerves were then cut so that the entire brain could be lifted from the skull. A single cut was performed down the interhemispheric fissure to separate both brain hemispheres. The tissues were placed into collection vials and frozen with liquid nitrogen. The samples were then placed into −80 °C freezers until the time of further dissection. Sub-dissection was performed on ice utilizing a Petri dish (Fisher 0875711Aa) as a platform. The cerebellum was dissected free first using a cut below the orbital region and one just above the brain stem. This tissue was then was placed into a collection vial and frozen on dry ice. Next the cerebral cortex was dissected free using a sweeping cut in a semicircular motion starting just above the corpus callosum. This motion was to make sure to keep the temporal region attached. The cerebral cortex was oriented toward the anterior and then cut directly in the middle, separating it into anterior and posterior portions. These regions were placed in collection vials and frozen on dry ice. Finally, the brain stem was separated from the midbrain based on its narrowing posterior to the midbrain. These sections were placed in collection vials and frozen on dry ice.

#### Genotyping

Genotyping was performed on ear-punch tissue samples from mice that were approximately 35 days old. 100 µL of genomic DNA was extracted from approximately 15 mg ear samples using the QIAmp Tissue DNA extraction protocol for the QIAcube HT automated liquid handler. gDNA quality and quantity were measured on a SpectraMax M5 plate reader taking readings at 260 nm and 280 nm. gDNA from the ear tissue of a verified high-copy SOD1 mouse was two-fold serially diluted to create a standard curve starting from 5 ng/uL; the concentration of the standard was verified using a SpectraMax NanoDrop. A relative quantification qPCR was used to probe for the human SOD1 gene, which is copy-number variable in the SOD1^G93A^ mouse model, using murine Gapdh as an endogenous control gene. The absolute concentration of the unknown samples was interpolated from the standard curve and the resulting concentrations were used to normalize the SOD1 Cts. The resulting Cts were plotted on a graph using GraphPad Prism and the mice that stood out with an elevated Ct were flagged as low-copy. The SOD1 primer/probe set was custom-made from Life Technologies using the following sequences: GTAAATCAGCTGTTTTCTTTGTTCAGA for the forward primer, TTCACTGGTCCATTACTTTCCTTTAA for the reverse primer, and ACTCTCTCCAACTTTG for the VIC probe. The Gapdh primer/probe set was a Life Technologies Assay-On-Demand with Assay ID # Mm00186822_cn.

#### Tissue Homogenization

Tissues were homogenized in ice-cold homogenization buffer consisting of 1X PBS and an EDTA-free Pierce protease inhibitor (Thermo Scientific, Cambridge, MA). One tablet of the protease inhibitor was used for each 50 mL of 1X PBS. For each sample, 30 µL of the homogenization buffer was used per 1 mg of tissue weight. Homogenization was achieved in 1.4 mm ceramic bead tubes (MO BIO, Carlsbad, CA) using a Precellys 24 homogenizer (5000 rpm, 2 × 20 sec). The homogenates were then split into two parts which were subsequently differentially processed depending on the nature of the experiments. Approximately 2/3 of each homogenate by volume was used for the filter trap assay, and the remaining 1/3 for the ELISA assay. For the filter trap assay, the homogenate was centrifuged at 800 × g for 10 min at 4 °C and for the ELISA assay the homogenate was centrifuged at 14,000 × g for 60 min at 4 °C. In both cases, after the centrifugation, the supernatants were collected, assayed for protein content using a Pierce BCA assay kit (Thermo Scientific, Cambridge, MA), and stored at −80 °C.

#### s-misSOD1 ELISA Standard Preparation (SOD1 Denaturation)

Superoxide dismutase from human erythrocytes was purchased from Sigma-Aldrich (Catalog Number S9636). The amount of lyophilized SOD1 protein in each vial was calculated based on the units of SOD1 indicated and the predetermined activity of each unit in a cytochrome c reduction assay for the particular batch (provided by the manufacturer). SOD1 was solubilized, using a 5 M guanidinium chloride and 25 mM EDTA solution, to a final concentration of 23 µM. This SOD1 solution was incubated overnight at room temperature while gently stirred on a sample mixer. The following day, it was injected into a 3,500 MWCO Slide-A-Lyzer Dialysis cassette (Thermo Scientific, Rockford, IL) which was then floated in a dialysis buffer containing 10 mM Hepes (pH 7.4), 150 nM NaCl, 3 mM EDTA, 0.05% Tween, for 5 hours at room temperature. The dialysis cassette was transferred into a beaker containing fresh dialysis buffer and it was left to float overnight at 4 °C. The presumed denatured SOD1 solution was extracted from the dialysis cassette using a 10 mL syringe and was centrifuged at 4000 x g for 5 min to remove SOD1 aggregates. The supernatant, containing denatured SOD1, was collected, aliquoted, and stored at −80 °C.

#### Filter Trap Assay for SOD1(+) Aggregates

A size exclusion filter trap assay was developed for the detection of human SOD1(+) aggregates in tissue lysates. Briefly, spinal cord or brain lysates were thawed and protein concentrations determined by BCA. The BioRad dotblot apparatus was used to filter dilutions of the lysates through a 0.2 micron cellulose acetate membrane (Advantec C020A330R). All steps were done at room temperature. Prior to assembly, the cellulose acetate membrane (9.5 mm × 13 mm) was soaked in PBS for 5 min. Lysate samples were diluted to final concentrations of approximately 100, 50, and 25 μg/200 μL in PBS/0.05% SDS. After dotblot apparatus assembly, the membrane was washed 2X with 200 uL/well PBS. Samples were loaded 200 uL/well leaving top and bottom rows empty; any empty wells received 200 μl PBS. All samples were run in duplicate blots. Vacuum was applied to filter samples through the membrane followed by 2 washes with PBS. After filtration, the membrane was removed and marked with pencil to keep the blot in this orientation for all incubations and washes. The blot was transferred to a 100 mm dish and incubated in Superblock buffer (Thermo. Sci. 37515) for 30 min. All antibody incubation and wash steps were done at room temperature on a standard analog shaker. One blot was incubated overnight with polyclonal rabbit anti-human SOD1 (Abcam ab52950) at a 1:2000 dilution in Superblock; the duplicate blot was incubated in Superblock only to determine background luminescence. The next day, blots were rinsed in tap water and washed (5X) in PBS/0.05% Tween 20 for 5 min. After the last wash, the blots were transferred to clean 100 mm plates. All blots were then incubated for 1 h with HRP-goat anti-rabbit IgG (Jackson Immuno. 111-035-144) at a concentration of 1:70,000 in Superblock/0.02% Tween 20 and covered to avoid exposure to light. The blots were washed 5X as above followed by 2X with PBS only. The blots were incubated with Chemiluminescent Western Blotting Substrate (Pierce ECL 32106) for 1 min without shaking and cut with a scalpel into vertical strips corresponding to vertical columns of wells from the dotblot. The strips were placed on top of wells on a 96-well opaque white plate (Greiner Lumitrac 600) leaving a space between each column, covered with an adhesive plate sealer (Thermo. AB-0558) and immediately read for luminescent signal on a SpectraMax M5 plate reader.

#### Assay for Misfolded SOD1

Microtiter plates (Corning 3690; 96-well half area plates) were coated overnight at 4 °C with a recombinant human-derived monoclonal anti-misfolded human SOD1 antibody NI-204.B (courtesy of Neurimmune) at 10 μg/mL in G-PBS (Gibco 10010-031). Plates were rinsed and blocked with PBS/1% BSA for 1 h at room temperature. Lysate sample dilutions were prepared in assay buffer (PBS/0.5% BSA/0.1% Tween 20) and tested in duplicate. A standard curve of denatured human erythrocyte derived SOD1 (see preparation of denatured SOD1 above) was included with serial two-fold dilutions from 0.3 to 0.0003 μg/mL as well as background control wells with assay buffer only. Samples and standards were incubated for 1 h at room temperature. Plates were then washed with PBS/0.1% Tween 20 and incubated with rabbit monoclonal anti-human SOD1 (Abcam Ab79390) at a 1:1000 dilution in assay buffer for 1 h at room temperature. Following another set of washes, plates were incubated with polyclonal biotinylated - goat anti-rabbit IgG (Jackson Immuno. 111-065-144) at a 1:20,000 dilution in assay buffer. After 1 h plates were washed and incubated for an additional hour with a 1:10,000 dilution of Streptavidin Poly-HRP (Pierce 21140). After the last wash, 50 μL of TMB HRP substrate solution (Surmodics) was added to each well and color development was stopped with 25 μL per well of 2 N H2SO4 (R&D DY994). The optical density at 450 nM wavelength was read on a SpectraMax M5 plate reader.

#### SOD1 mRNA Expression

The SOD1 mRNA expression qPCR was performed on laterally bisected spinal cords and 15 mg tail tip samples from SOD1 mice. RNA was extracted using the Agencourt RNAdvance Tissue Kit for the Beckman Coulter BioMek FX^P^ automated liquid handler. RNA quality and quantity were measured using the SpectraMax NanoDrop. cDNA was synthesized using the Applied Biosystems High-Capacity cDNA Reverse Transcription kit. Normalization genes were selected using the Applied Biosystems Mouse Endogenous Control array card. Four control genes per tissue type were chosen; the genes chosen were those with the lowest variation in tissues across a range of SOD1 mouse ages and neurological scores. The spinal cord sample controls were murine Gapdh, Ppia, Tbp, and Ywhaz. The tail tip sample controls were murine Hprt, Polr2a, Ppia, and Ywhaz. A relative quantification qPCR was run using the Applied Biosystems 7900HT qPCR platform using SOD1 (Assay ID Hs00533490_m1, Thermo Fisher Scientific) as the target gene. The resulting Cts were normalized using the control genes and GeNorm software to produce relative SOD1 expression values for each sample.

#### Immunohistochemistry

Frozen spinal cord tissue section slides were equilibrated to room temperature with prewarmed PBS then rinsed with PBS/0.1% Triton X-100. Sections were incubated with blocking buffer with 1% BSA and 2% normal donkey serum with 0.1% Triton X-100 followed by primary antibodies diluted in blocking buffer. After incubation with the primary antibodies, slides were rinsed in PBS/0.1% Triton X-100 and incubated in species specific AF488 or AF594-conjugated secondary antibodies. Slides were coverslipped with glass slides using aqueous mounting medium with Dapi (Vectashield). Images were collected on a Zeiss LSM 700 Laser Scanning Microscope at 10X and 20X magnification. Using Fiji, high-resolution three-dimensional images were generated from serial optical sections.

#### Quantification and Statistical Analysis

All statistical analyses were conducted using GraphPad Prism 6 for Mac OS. P-values and R-squared presented in Figs 3 and 4 were derived from linear regression analyses. Intergroup comparisons of mean values for all groups were conducted using one-way ANOVA followed by the Holm-Šidák test, a method which corrects for multiple comparisons.

#### Key Resources Table

B6SJL-Tg(SOD1*G93A)1Gur Mice.

α-miSOD1- anti-misfolded SOD1 antibody used for ELISA.

NI204.P used for immunofluorescence imaging.

Human erythrocyte derived SOD1 Sigma-Aldrich S9636.

0.2 micron cellulose acetate membrane Advantec C020A330R.

Polyclonal rabbit anti-human SOD1 Abcam ab52950.

HRP-goat anti-rabbit IgG Jackson Immuno. 111-035-144.

Rabbit monoclonal anti-human SOD1 Abcam ab79390.

Polyclonal biotinylated – goat anti-rabbit IgG Jackson Immuno. 111-065-144.

Steptavidin Poly-HRP Pierce 21140.

SOD1 PCR Assay ID Hs00533490_m1, ThermoFisher Scientific.

## Supplementary information

Supplementary Information
