# Supplementary material for: SOD1-positive aggregate accumulation in the CNS predicts slower disease progression and increased longevity in a mutant SOD1 mouse model of ALS
Source: Sci Rep. 2019 Apr 30;9:6724. doi: 10.1038/s41598-019-43164-z (PMC6491559; doi:10.1038/s41598-019-43164-z)
Supplement: Supplementary file 1 — Supplementary Information [file 41598_2019_43164_MOESM1_ESM.pdf]

SOD1-positive aggregate accumulation in the CNS predicts slower disease progression and increased longevity in a mutant SOD1 mouse model of ALS.

Cindy Gill, James P. Phelan, Theo Hatzipetros, Joshua D. Kidd, Valerie R. Tassinari, Beth Levine, Monica Z. Wang, Andrew Moreno, Kenneth Thompson, Marcel Maier, Jan Grimm, Alan Gill, Fernando G. Vieira

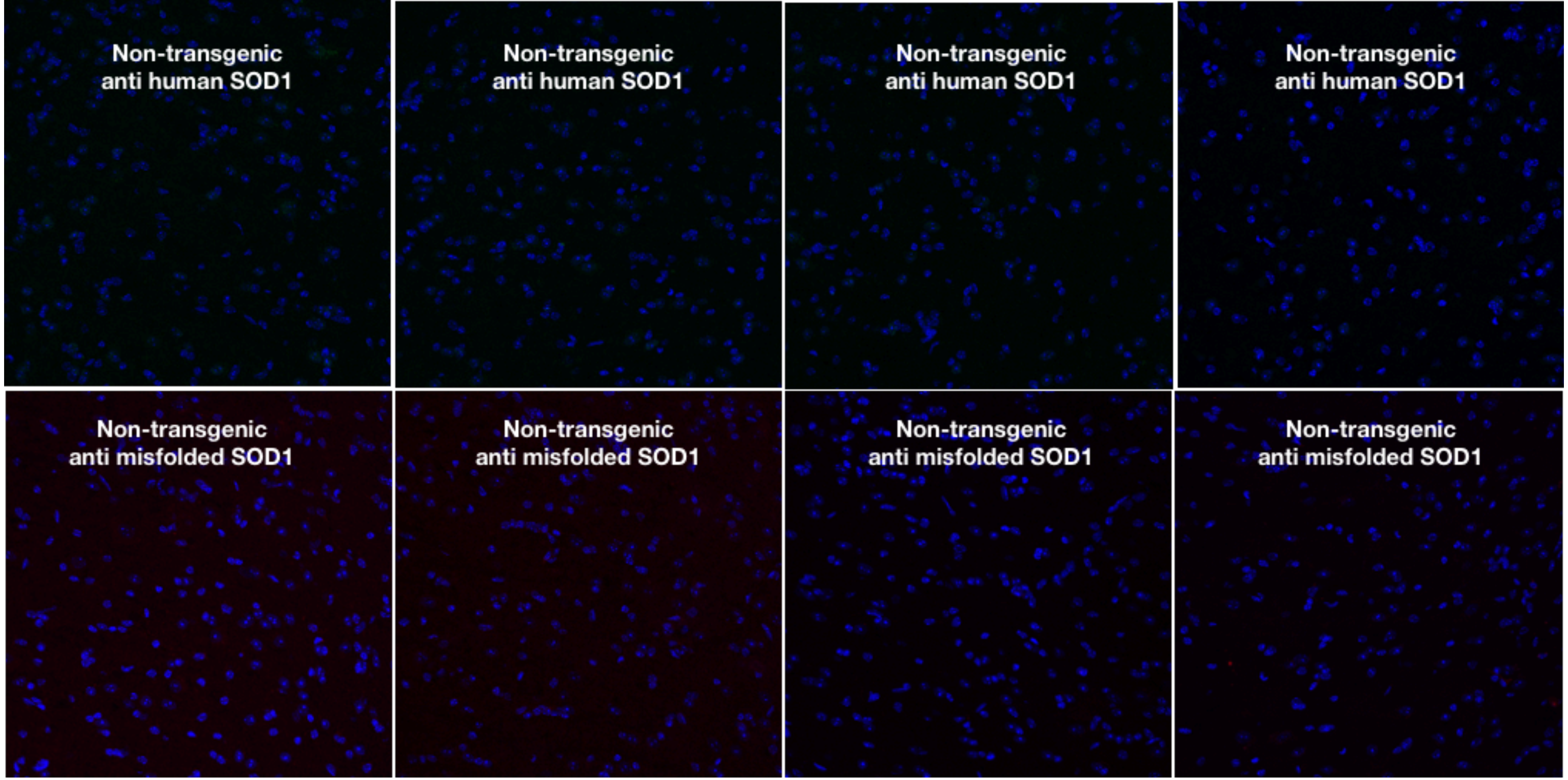

Supplemental Figure 1: Non-transgenic lumbar spinal cord samples stained for both human SOD1 and misfolded human SOD1. Each field presents an individual mouse

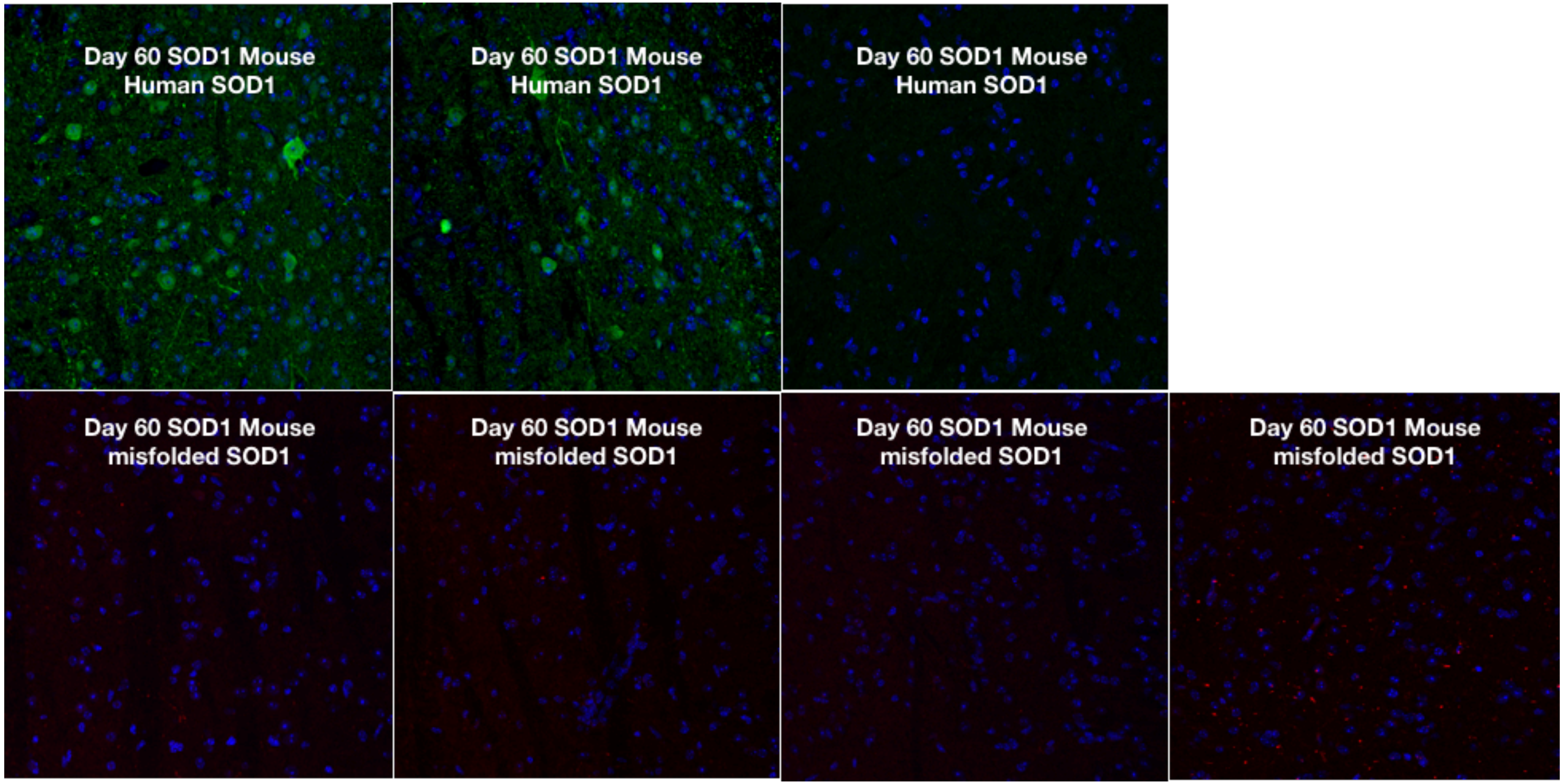

Supplemental Figure 2: P60 G93A-SOD1 mouse lumbar spinal cord samples stained for both human SOD1 and misfolded human SOD1. Each field presents an individual mouse

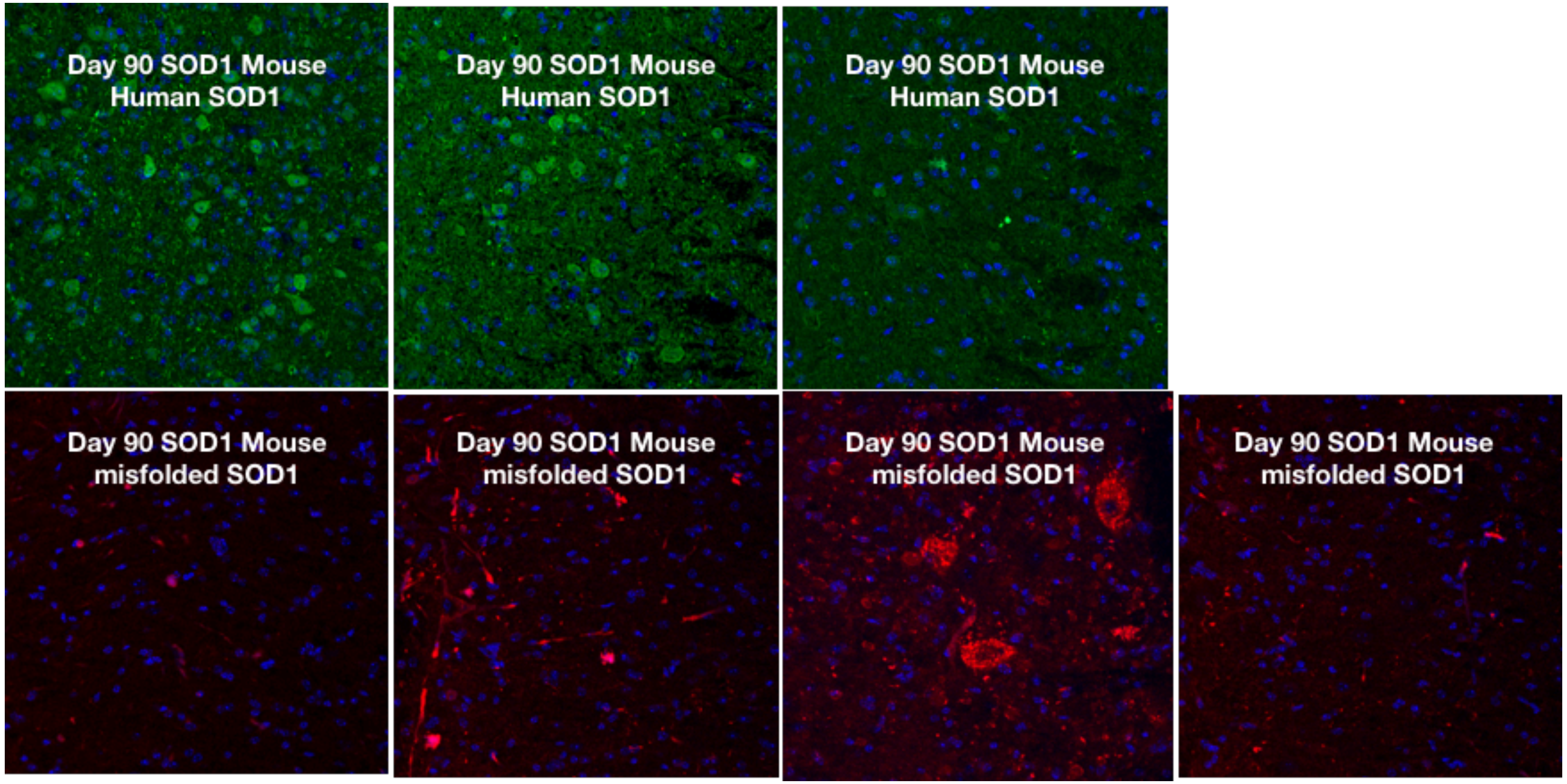

Supplemental Figure 3: P90 G93A-SOD1 mouse lumbar spinal cord samples stained for both human SOD1 and misfolded human SOD1. Each field presents an individual mouse

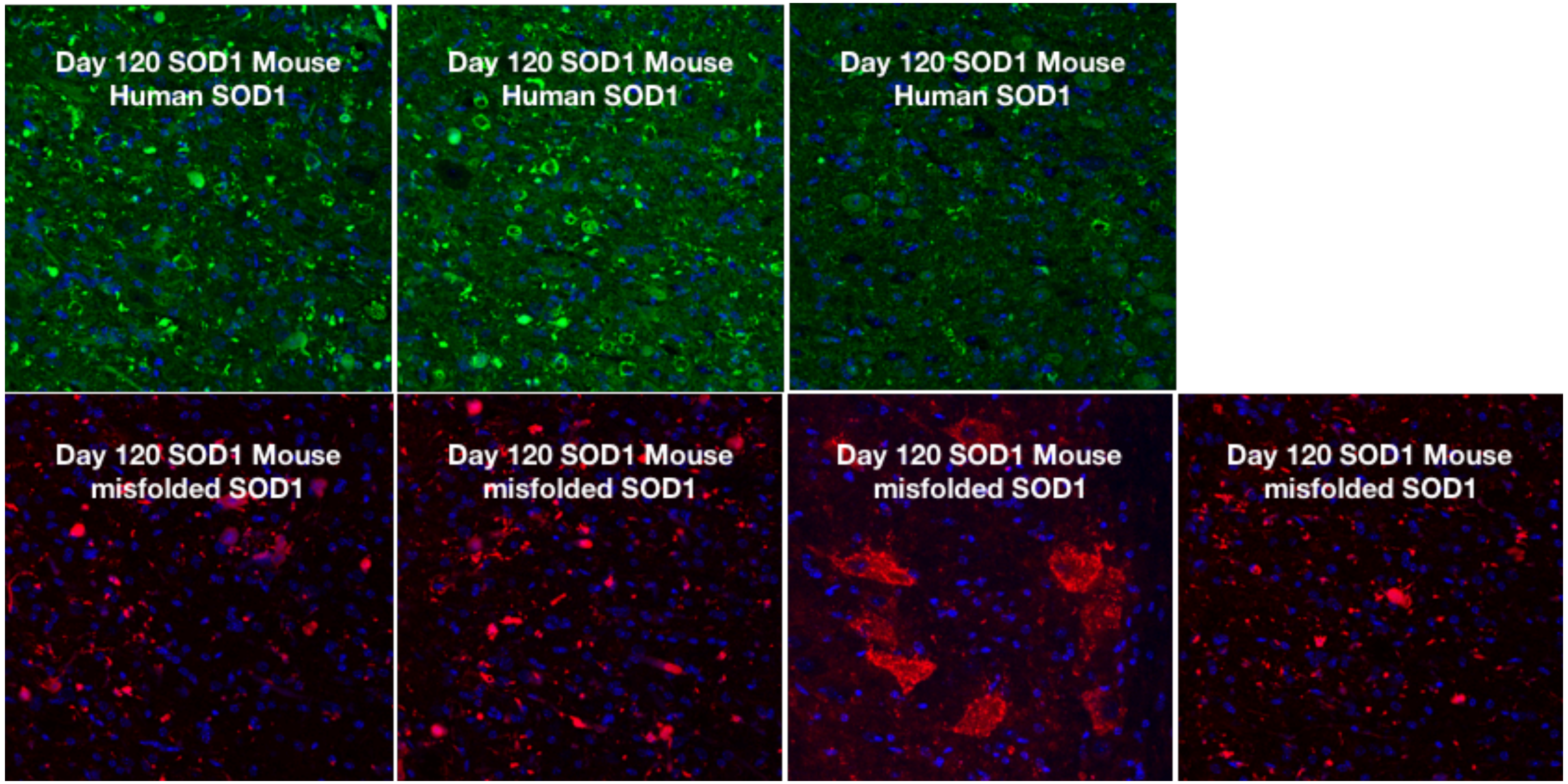

Supplemental Figure 4: P120 G93A-SOD1 mouse lumbar spinal cord samples stained for both human SOD1 and misfolded human SOD1. Each field presents an individual mouse

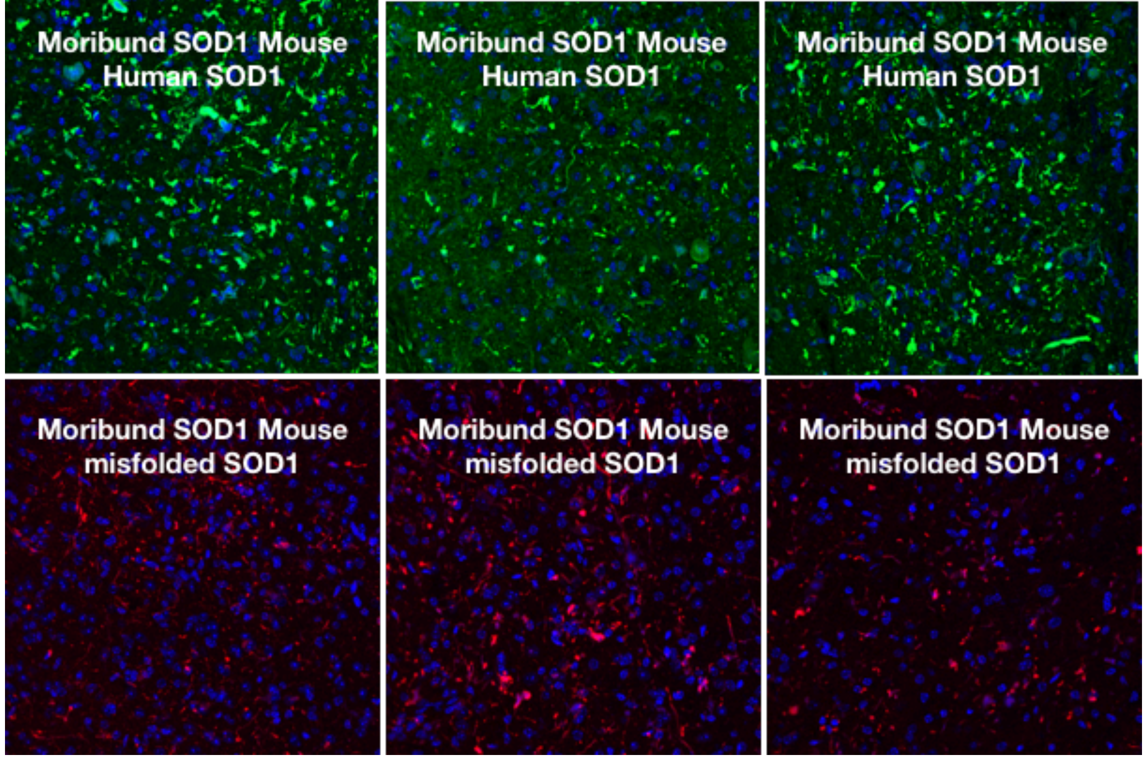

Supplemental Figure 5: Moribund G93A-SOD1 mouse lumbar spinal cord samples stained for both human SOD1 and misfolded human SOD1. Each field presents an individual mouse

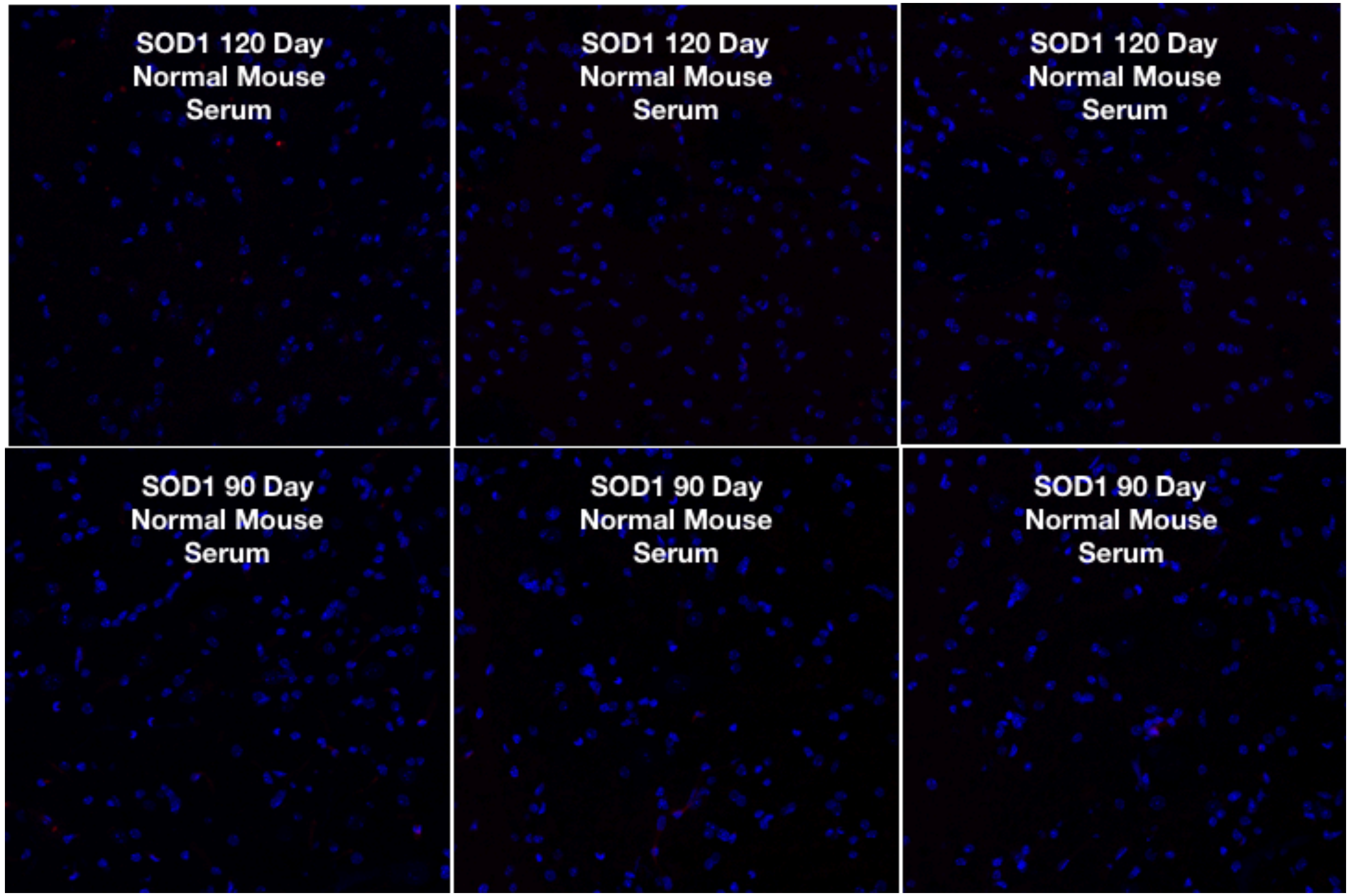

Supplemental Figure 6: P90 and 120 G93A-SOD1 mouse lumbar spinal cord stained with normal rat serum and secondary antibody as background controls. Each field presents an individual mouse

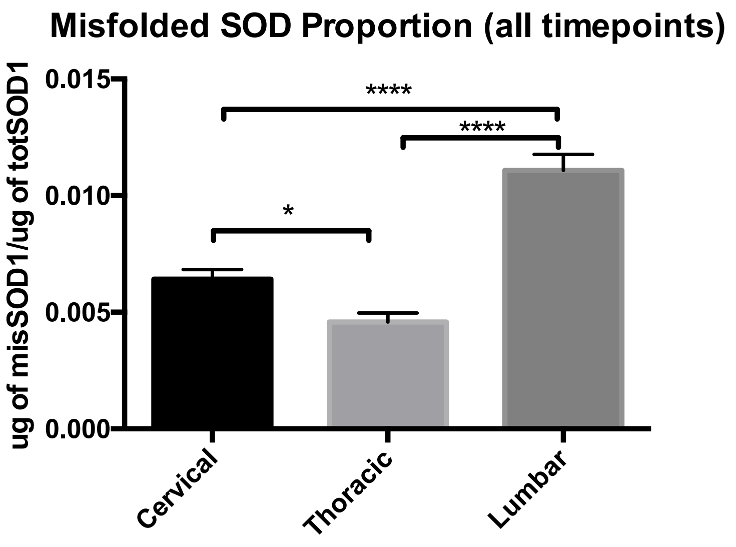

Supplemental Figure 7: The mean proportions of total SOD1 that is misfolded in cervical, thoracic, and lumbar spinal cords of G93A-SOD1 mice.

Supplemental Table 1. Anterior Cortex

Holm-Sidak's Multiple Comparisons Test

Comparison	Mean Diff.	Adjusted P Value	Significant?	Summary
d70 vs. d100	-0.692	0.3514	No	ns
d70 vs. d120	-0.898	0.2391	No	ns
d70 vs. d130	-2.307	0.0038	Yes	**
d100 vs. d120	-0.206	0.6506	No	ns
d100 vs. d130	-1.615	0.0232	Yes	*
d120 vs. d130	-1.410	0.0310	Yes	*

Supplemental Table 2. Cerebellum

Holm-Sidak's Multiple Comparisons Test

Comparison	Mean Diff.	Adjusted P Value	Significant?	Summary
d70 vs. d100	2.097	0.6268	No	ns
d70 vs. d120	-0.836	0.6268	No	ns
d70 vs. d130	-2.310	0.6063	No	ns
d100 vs. d120	-2.933	0.4201	No	ns
d100 vs. d130	-4.407	0.1508	No	ns
d120 vs. d130	-1.474	0.6268	No	ns

Supplemental Table 3. Midbrain

Holm-Sidak's Multiple Comparisons Test

Comparison	Mean Diff.	Adjusted P Value	Significant?	Summary
d70 vs. d100	-0.390	0.8981	No	ns
d70 vs. d120	-2.261	0.7994	No	ns
d70 vs. d130	-13.000	0.0083	Yes	**
d100 vs. d120	-1.871	0.7994	No	ns
d100 vs. d130	-12.610	0.0085	Yes	**
d120 vs. d130	-10.740	0.0094	Yes	**

Supplemental Table 4. Brainstem

Holm-Sidak's Multiple Comparisons Test

Comparison	Mean Diff.	Adjusted P Value	Significant?	Summary
d70 vs. d100	-4.674	0.7248	No	ns
d70 vs. d120	-25.130	0.2372	No	ns
d70 vs. d130	-34.330	0.1210	No	ns
d100 vs. d120	-20.460	0.2652	No	ns
d100 vs. d130	-29.660	0.1389	No	ns
d120 vs. d130	-9.199	0.6807	No	ns

Supplemental Table 5. Cervical Spinal Cord

Holm-Sidak's Multiple Comparisons Test

Comparison	Mean Diff.	Adjusted P Value	Significant?	Summary
d70 vs. d100	-0.333	0.9359	No	ns
d70 vs. d120	-1.505	0.9359	No	ns
d70 vs. d130	-10.710	0.0019	Yes	**
d100 vs. d120	-1.173	0.9359	No	ns
d100 vs. d130	-10.380	0.0030	Yes	**
d120 vs. d130	-9.208	0.0065	Yes	**

Supplemental Table 6. Thoracic Spinal Cord

Holm-Sidak's Multiple Comparisons Test

Comparison	Mean Diff.	Adjusted P Value	Significant?	Summary
d70 vs. d100	0.185	0.8117	No	ns
d70 vs. d120	-0.502	0.7670	No	ns
d70 vs. d130	-7.488	< 0.0001	Yes	****
d100 vs. d120	-0.687	0.7582	No	ns
d100 vs. d130	-7.672	< 0.0001	Yes	****
d120 vs. d130	-6.985	< 0.0001	Yes	****

Supplemental Table 7. Lumbar Spinal Cord

Holm-Sidak's Multiple Comparisons Test

Comparison	Mean Diff.	Adjusted P Value	Significant?	Summary
d70 vs. d100	-0.333	0.9359	No	ns
d70 vs. d120	-1.505	0.9359	No	ns
d70 vs. d130	-10.710	0.0019	Yes	**
d100 vs. d120	-1.173	0.9359	No	ns
d100 vs. d130	-10.380	0.0030	Yes	**
d120 vs. d130	-9.208	0.0065	Yes	**

Supplemental Table 8. All Tissue Types, All Time Points Pooled

Holm-Sidak's Multiple Comparisons Test

Comparison	Mean Diff.	Adjusted P Value	Significant?	Summary
Anterior Cortex vs. Cerebellum	-2.525	0.9851	No	ns
Anterior Cortex vs. Midbrain	-4.229	0.9230	No	ns
Anterior Cortex vs. Brain Stem	-24.880	< 0.0001	Yes	****
Anterior Cortex vs. Cervical Spinal Cord	-5.262	0.4059	No	ns
Anterior Cortex vs. Thoracic Spinal Cord	-1.661	0.9851	No	ns
Anterior Cortex vs. Lumbar Spinal Cord	-3.086	0.9409	No	ns
Cerebellum vs. Midbrain	-1.704	0.9888	No	ns
Cerebellum vs. Brain Stem	-22.35	< 0.0001	Yes	****
Cerebellum vs. Cervical Spinal Cord	-2.738	0.9626	No	ns
Cerebellum vs. Thoracic Spinal Cord	0.864	0.9888	No	ns
Cerebellum vs. Lumbar Spinal Cord	-0.561	0.9888	No	ns
Midbrain vs. Brain Stem	-20.65	< 0.0001	Yes	****
Midbrain vs. Cervical Spinal Cord	-1.034	0.9888	No	ns
Midbrain vs. Thoracic Spinal Cord	2.568	0.9700	No	ns
Midbrain vs. Lumbar Spinal Cord	1.143	0.9888	No	ns
Brain Stem vs. Cervical Spinal Cord	19.62	< 0.0001	Yes	****
Brain Stem vs. Thoracic Spinal Cord	23.22	< 0.0001	Yes	****
Brain Stem vs. Lumbar Spinal Cord	21.79	< 0.0001	Yes	****
Cervical Spinal Cord vs. Thoracic Spinal Cord	3.602	0.4165	No	ns
Cervical Spinal Cord vs. Lumbar Spinal Cord	2.177	0.9409	No	ns
Thoracic Spinal Cord vs. Lumbar Spinal Cord	-1.425	0.9851	No	ns
